# Aqueous Extract of Shi-Liu-Wei-Liu-Qi-Yin Induces G2/M Phase Arrest and Apoptosis in Human Bladder Carcinoma Cells via Fas and Mitochondrial Pathway

**DOI:** 10.1093/ecam/nep016

**Published:** 2011-01-03

**Authors:** Ting-Tsz Ou, Chau-Jong Wang, Guang-Uei Hung, Cheng-Hsun Wu, Huei-Jane Lee

**Affiliations:** ^1^Institute of Biochemistry and Biotechnology, College of Medicine, Chung Shan Medical University, Taichung, Taiwan; ^2^Department of Nuclear Medicine, Chang Bing, Show-Chwan Memorial Hospital, Changhua, Taiwan; ^3^Department of Anatomy, China Medical University, No. 91, Hsueh-Shih Road, Taichung, Taiwan

## Abstract

Shi-Liu-Wei-Liu-Qi-Yin (SLWLQY) was traditionally used to treat cancers. However, scientific evidence of the anticancer effects still remains undefined. In this study, we aimed to clarify the possible mechanisms of SLWLQY in treating cancer. We evaluated the effects of SLWLQY on apoptosis-related experiments inducing in TSGH-8301 cells by (i) 3-(4,5-dimethylthiazol-zyl)-2,5-diphenylterazolium bromide (MTT) for cytotoxicity; (ii) cell-cycle analysis and (iii) western blot analysis of the G2/M-phase and apoptosis regulatory proteins. Human bladder carcinoma TSGH-8301 cells were transplanted into BALB/c nude mice as a tumor model for evaluating the antitumor effect of SLWLQY. Treatment of SLWLQY resulted in the G2/M phase arrest and apoptotic death in a dose-dependent manner, accompanied by a decrease in cyclin-dependent kinases (cdc2) and cyclins (cyclin B1). SLWLQY stimulated increases in the protein expression of Fas and FasL, and induced the cleavage of caspase-3, caspase-9 and caspase-8. The ratio of Bax/Bcl_2_ was increased by SLWLQY treatment. SLWLQY markedly reduced tumor size in TSGH-8301 cells-xenografted tumor tissues. In the tissue specimen, SLWLQY up-regulated the expression of Fas, FasL and Bax proteins, and down-regulated Bcl_2_ as well as in *in vitro* assay. Our results showed that SLWLQY reduced tumor growth, caused cell-cycle arrest and apoptosis in TSGH-8301 cells via the Fas and mitochondrial pathway.

## 1. Introduction

Oriental herbal medicines have been used since ancient times to treat malignancies. Systematic characterization of active phytochemicals in medicinal herbs and their mechanisms of action are important for providing the rationale for their efficacy and for transforming herbal practices into evidence-based medicine [1]. Typical traditional Chinese medicines consist of 5–16 components that are mixed to minimize side effects and maximize medical effects. Recently, there has been increasing interest in the biological activity of traditional medicines, and numerous studies support their potential clinical benefit for diseases that are difficult to treat such as cancer [2–5]. Shi-Liu-Wei-Liu-Qi-Yin (SLWLQY) formula is a prescription for patients with various tumors and is composed of 16 oriental medicinal herbs such as *Panax Ginseng* C.A., *Astragalus Membranaceus*, *Paeonia lactiflora* Pall, *Angelica Sinensis* Diels, and so forth (Table 1).

The majority of bladder cancers (90%) are transitional cell carcinoma (TCC). Transitional cell carcinoma, occurring in the renal pelvis, ureter, urinary bladder and urethra, is the most serious cancer of the urinary tract [6]. Recent studies have begun to elucidate the underlying genetic determinations of the morphologic and biologic characteristics of these different forms of bladder cancer [7]. The molecular alterations that precede morphologic changes and are responsible for tumorigenesis and progression of bladder cancer also include alterations in cell-cycle regulators causing uncontrolled cancer growth [8]. In general, the progression of cell cycle is a complex process involving resting G0 phase, and cell growth involving G1, S and G2/M phase in eukaryotes [9]. The cell-cycle phases receive different growth controlling signals that are processed for the activation of different members of the cyclin-dependent kinases (CDKs) [10, 11]. Cyclin-dependent kinases are governed by their regulatory subunits known as cyclins, and are activated at a specific phase of the cell cycle.

In this study, we aimed to study new approaches that could alter uncontrolled human bladder TCC growth by modulating cell-cycle regulators causing cell-cycle arrest and could be useful in human bladder cancer prevention. We assessed the efficacy of SLWLQY on cell growth, cell-cycle progression and apoptotic death in human bladder TCC cell-line-TSGH-8301. The results obtained clearly demonstrate that SLWLQY inhibits human bladder TCC cell growth, causes cell-cycle arrest and apoptosis, and that a strong decrease in both Cdc2 and cyclinB1 levels are responsible for the observed effects of SLWLQY in cell-cycle arrest. Furthermore, SLWLQY caused caspase-3, caspase-9 and caspase-8 cleavages contribute to its apoptotic response in TSGH-8301 cells.

## 2. Methods

### 2.1. Reagents

Tris-HCl, EGTA, SDS, bovine serum albumin (BSA), Nonidet P-40, dimethyl sulfoxide (DMSO), RNase A and polyclonal antibody against *α*-actin were purchased from Sigma Chemical Co. (St Louis, MO). Minimum essential medium (MEM), Dulbecco's modified Eagle medium (DMEM), RPMI medium 1640, F-12 nutrient mixture, fetal bovine serum (FBS), penicillin–streptomycin mixed antibiotics, l-glutamine, Dulbecco`s phosphate buffer solution (PBS) and trypsin–EDTA, were purchased from Gibco/BRL (Gaithersburg, MD). Antibodies against Bcl-2, Bax, caspase 3, caspase 8, Fas, fasL, cyclinB1 and Cdc2 were from Santa Cruz (Santa Cruz, CA). The enhanced chemiluminescence (ECL) kit was purchased from Amersham Life Science (Amersham, UK).

### 2.2. Preparation of SLWLQY Extract

The SLWLQY prescription consists of 16 different medical plant ingredients as shown in Table 1. All of the herbs were purchased from Changhwa in Taiwan. To prepare the aqueous extract of SLWLQY, 455 g of dried materials was extracted with 3 l of boiling water for 2 h. The prepared extract was freeze-dried and dissolved in distilled water, filtered and stored at −20°C until use. The average yield of dried extract was about 16.3%.

### 2.3. HPLC Assay for SLWLQY

The components of SLWLQY were determined by HPLC analysis using a Hewlett-Packard Vectra 436/33 N system with a diode array detector. The HPLC method employed a 5 *μ*m RP-18 column (4.6 × 150 mm i.d.). The SLWLQY were filtered through a 0.45 *μ*m filter disc and 10 *μ*l were injected onto the column. The chromatography was monitored at 280 nm, and UV spectra were collected to confirm peak purity. The mobile phase contained two solvents (A, formic acid/water = 10 : 90; B, formic acid/water/acetonitrile = 10 : 60 : 30) run by a linear gradient method at room temperature as follows: from 20% B to 85% B (flow rate = 0.8 ml min^−1^) over 55 min.

### 2.4. Cell Line and Cell Culture

Human urinary bladder cancer cells (TSGH-8301) were maintained in RPMI 1640 medium. TSGH-8031 cells were derived from a well-differentiated human TCC of the urinary bladder [12], having wt p53 but mutant Rb gene. Human gastric carcinoma (AGS) were maintained in F-12 nutrient mixture medium; adenocarcinoma MCF-7 and human oral epidermoid carcinoma KB were maintained in MEM; and hepatocarcinoma Huh-7 and human fetal liver cells WRL-68 were maintained in Dulbecco's modified Eagle's medium. Cells were cultured at 37°C in 5% CO_2_ in medium supplemented with 10% FBS and antibiotics (100 U ml^−1^ of penicillin and 100 *μ*g ml^−1^ of streptomycin). All of the cell lines were purchased from BCRC, Hsinchu, Taiwan.

### 2.5. Assessment of Cell Viability

Cells were seeded at a density of 1 × 10^5^ cells ml^−1^ and incubated with SLWLQY at various concentrations (0–5 mg ml^−1^) for 24 h. Thereafter, the medium was changed and 3-(4,5-dimethylthiazol-zyl)-2, 5-diphenylterazolium bromide (MTT, 0.5 mg ml^−1^) was added to incubate for 4 h. The viable cell was directly proportional to the production of formazan. Following dissolution in isopropanol, the result was read at 563 nm with a spectrophotometer (Beckman DU640).

### 2.6. Flow Cytometry Analysis

Cell-cycle analysis was performed with a flow cytometer (FACS Calibur; BD Bioscience, CA, USA). Cells were cultured in 6 cm culture plates and treated with various concentrations of SLWLQY for 24 h. Thereafter, cells were washed twice with cold PBS solution. Then the cell suspension was centrifuged at 1500 g for 5 min, fixed and permeabilized with 70% ethanol at −20°C overnight. Prior to the samples being analyzed by the flow cytometry, 1 ml of cold propidium iodide (PI) stain solution (20 *μ*g ml^−1^ PI, 20 *μ*g ml^−1^ RNase A and 0.1% Triton X-100) was added to the mixture and it was incubated for 15 min in darkness at room temperature. Data acquisition and analysis were performed in the flow cytometer with accompanying software (CellQuest; BD Bioscience, CA). The percentage of hypodiploid cells (sub G1 phase) over total cells was calculated and represented as percent of apoptosis.

### 2.7. Western Blot Analysis

After treatment with the desired concentration of the SLWLQY for 24 h, the medium was removed and rinsed with PBS at room temperature. Then 0.5 ml of cold RIPA buffers (1% NP-40, 50 mM Tris–base, 0.1% SDS, 0.5% deoxycholic acid, 150 mM NaCl, pH 7.5) with fresh protease inhibitor was added. Cells were scraped and the lysate was centrifuged at 10 000 g for 10 min. Cell lysate (50 *μ*g) was mixed with an equal volume of electrophoresis sample buffer and then boiled for 10 min, followed by analysis using SDS–PAGE and transfer of protein was from the gel to nitrocellulose membrane (Millipore, Bedford, MA) by using electroblotting apparatus. Then the proteins were added with the ECL Western blotting detection reagents (Amersham Biosciences, USA) and analyzed using the Fui LAS-3000 imaging system (Japan). The antibodies used in this study included caspase-3, caspase-8, Bax, Bcl-2, Fas and FasL (Santa Cruz Biotech) anti-Cyclin B1 and anti-Cdc2 (Upstate), and anti-*β*-actin (Sigma Chemical Co).

### 2.8. Preparation for Cytosolic and Mitochondrial Fraction

Subcellular fraction was isolated by using Mitochondria Isolation Kit (Pierce, USA) to separate mitochondrion from cytosolic components. Cytochrome *c* existing in the two fractions was analyzed by western blot.

### 2.9. siRNA Transfection

RNA interference of Fas was performed using 19 bp siRNA duplexes purchased from Dharmacon (Thermo Scientific). The coding strand for Fas siRNA was GAACAUGGAAUCAUCAAGG. For transfection, TSGH-8301 cells were seeded in 6-well plates and transfected at 30% confluence with 100 nM siRNA duplexes using Lipofectamine (Invitrogen) according to the manufacturer`s recommendations. Cells were trasfected with target-specific Fas siRNA duplexes and control nonspecific siRNA duplex (Dharmacon) at the same final concentration (100 nM). After 12 h, cells were treated with SLWLQY (3 mg ml^−1^) for 24 h. Fas gene silencing was confirmed by western blot analysis of Fas protein expression.

### 2.10. Treatment of TSGH-8301 Cells Xenografts In Vivo

All animal experimental protocol used in this study was approved by the Institutional Animal Care and Use Committee of the Chung Shan Medical University (IACUC, CSMC), Taichung, Taiwan. Male BALB/c-nu mice were purchased from the National Laboratory Animal Center of Taiwan. They were maintained under Specific Pathogen Free conditions and supplied with sterilized food and water. After trypsinization, TSGH-8301 cells (1 × 10^7^ per 0.2 ml) were injected subcutaneously into the flanks of BALB/c-nu mice (4–6 weeks old, 18–20 g). One week later, the mice were divided randomly into four groups of four mice each. Mice were treated daily with 0.2 ml saline as the control or treated with the SLWLQY at doses of 0.2 g kg^−1^, 0.5 g kg^−1^ and 1 g kg^−1^ by using a gavage tube for 30 consecutive days. To monitor the drug toxicity, body weights of mice were measured each week. Thirty days later, the mice were sacrificed, and the tumor was removed, weighed and collected to analyze the protein expression.

## 3. Results

### 3.1. Composition of SLWLQY

**Figure 1:**
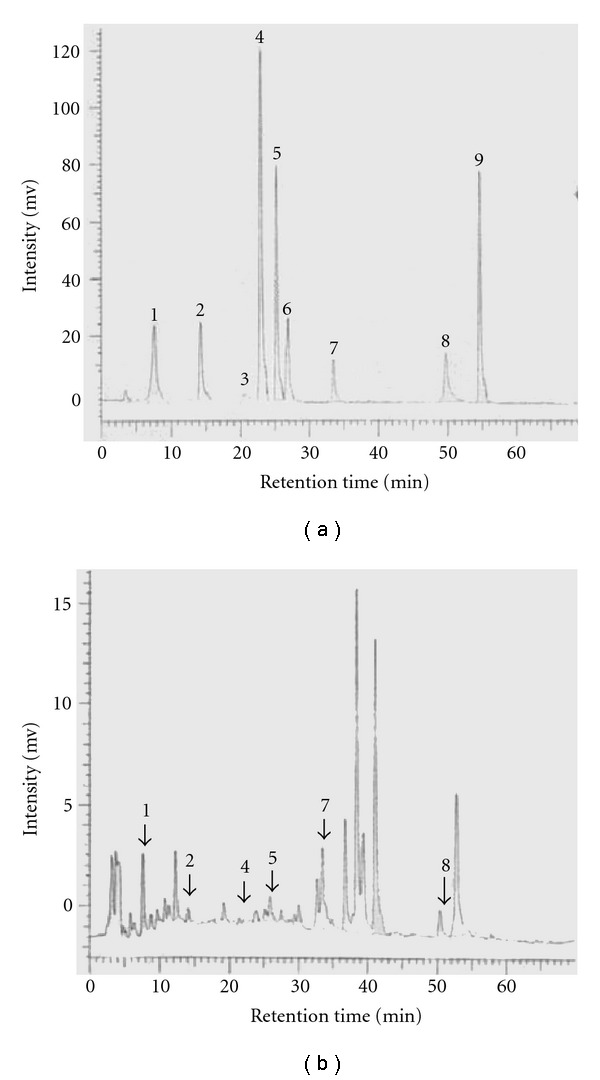
(a) HPLC chromatogram of nine kinds of standard polyphenols (10 mg ml^−1^). Peak: 1, gallic acid; 2, protocatechuic acid (PCA); 3, catechin; 4, epigallocatechin gallate (EGCG); 5, caffeic acid; 6, epicatechin; 7, rutin; 8, quercetin; 9, narigenin. (b) HPLC chromatograms of free polyphenols from SLWLQY extract (10 mg ml^−1^). Phenolic compounds correspond to peaks 1, 2, 4, 5, 6, 7 and 8 as in (a). Detector was set at 280 nm. The arrow indicated the retention time (RT) of merged both SLWLQY and different standards.

To establish the composition of SLWLQY, the contents of polyphenol were assayed. HPLC analysis of nine kinds of standard polyphenols showed the retention times (RT) of gallic acid, protocatechuic acid (PCA), catechin, epigallocatechin gallate (EGCG), caffeic acid, epicatechin, rutin, quercetin and naringenin were 7.68, 14.26, 20.69, 22.95, 25.24, 26.90, 33.53, 49.73 and 54.67 min, respectively (Figure 1). For the standardization of SLWLQY, the presence of gallic acid (2.28%), PCA (0.4%), EGCG (0.46%), caffeic acid (1.46%), rutin (6.71%) and quercetin (1.43%) contained in the SLWLQY. The composition of the SLWLQY used for the experiments was shown in Table 2. Phenolic compounds have long been recognized as potent antioxidants [13]. The three herbs of SLWLQY containing the highest phenolic compounds were *Paeonia lactiflora* Pall (gallic acid: 46.9%), *Glycyrrhiza Uralensis* Fish (rutin: 60.0%) and *Linderae Radix* (EGCG: 68.6%) in our study (data not shown).

### 3.2. Cytotoxicity of SLWLQY on Cancer Cells

**Figure 2:**
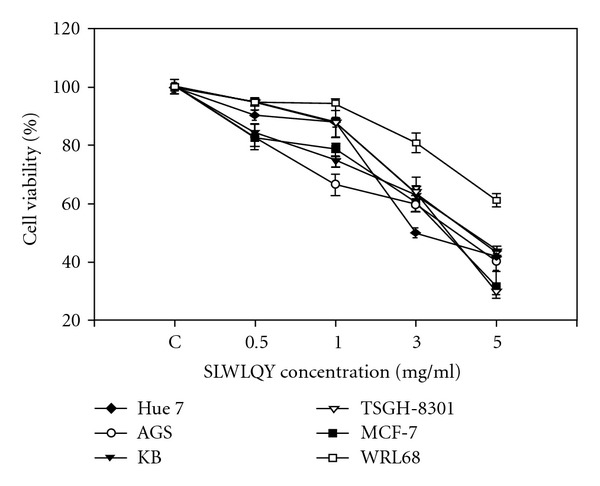
Cell viability of various cells (Huh-7, AGS, KB, MCF-7, WRL-68 and TSGH-8301 cells) treated with SLWLQY. Cells were treated with or without SLWLQY under different concentrations (0.5–5 mg ml^−1^) for 24 h. The number of viable cells in each well was quantified by using MTT assay. The results were represented as mean ± SD, *n* = 3.

Cell viability was assayed in cultures exposed to 0.5–5 mg ml^−1^ SLWLQY for 24 h. SLWLQY showed an inhibitory effect on the growth of Huh-7, AGS, KB, TSGH-8301, MCF-7 and WRL68 cells. SLWLQY exhibited the strongest potency of cytotoxicity in TSGH-8301 and MCF-7 cells at a dose of 5 mg ml^−1^. However, the death rate of WRL-68 cells (normal human liver cell line) was lower than that of TSGH-8301 cells, indicating that SLWLQY is less cytotoxic to normal cells (Figure 2).

### 3.3. Effect of SLWLQY on Inducing Apoptosis

**Figure 3:**
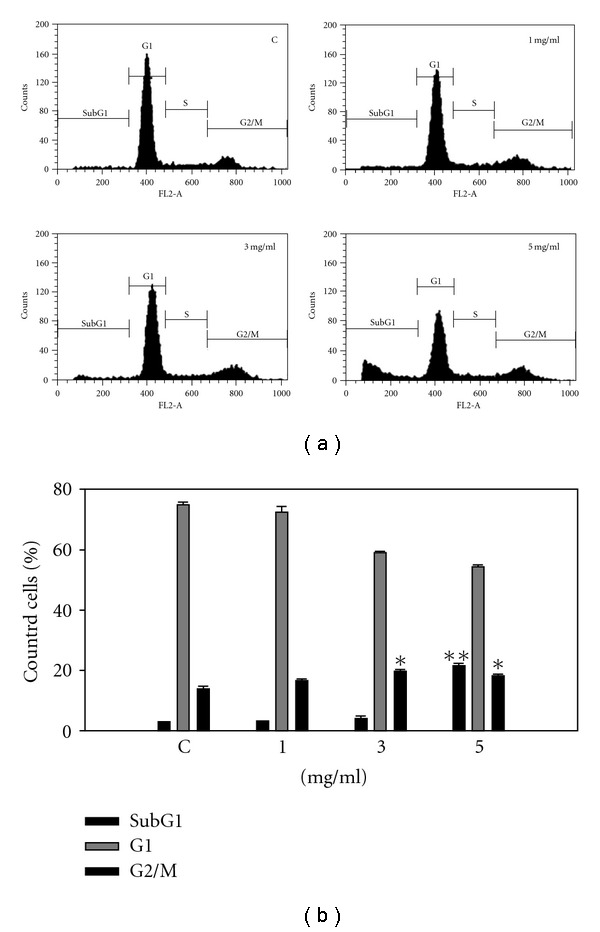
Apoptosis effects of SLWLQY on TSGH-8301 cells. (a) Cells were treated with 0 (control), 1, 3 and 5 mg ml^−1^ of SLWLQY for 24 h and subjected to flow cytometric analysis after PI staining. (b) Quantitative assessment of the percentage of TSGH-8301 cells in cell-cycle patterns (sub G1, G0/G1 and G2/M phase), as treated by SLWLQY, and represents the average of three independent experiments ± SD. *P < 0.05, **P < .01.

To examine whether the cytotoxicity of SLWLQY was due to the induction of apoptosis, cell-cycle analysis was performed by flow cytometry. After SLWLQY treatment for 24 h, TSGH-8301 cells increased DNA contents of the G2/M phase at the concentration of 3 mg ml^−1^, as compared with vehicle controls (13.85 ± 1.06% versus 19.60 ± 0.74%). We also found that the apoptotic TSGH-8301 cells dramatically increased to a level of 22.6% of counted cells with treatment of 5 mg ml^−1^ SLWLQY for 24 h as compared to 2.9% of that of control cells (Figures 3(a) and 3(b)).

### 3.4. SLWLQY Decreases Protein Levels of G2 Regulatory CDKs and Cyclins in Human TSGH-8301 Cells

**Figure 4:**
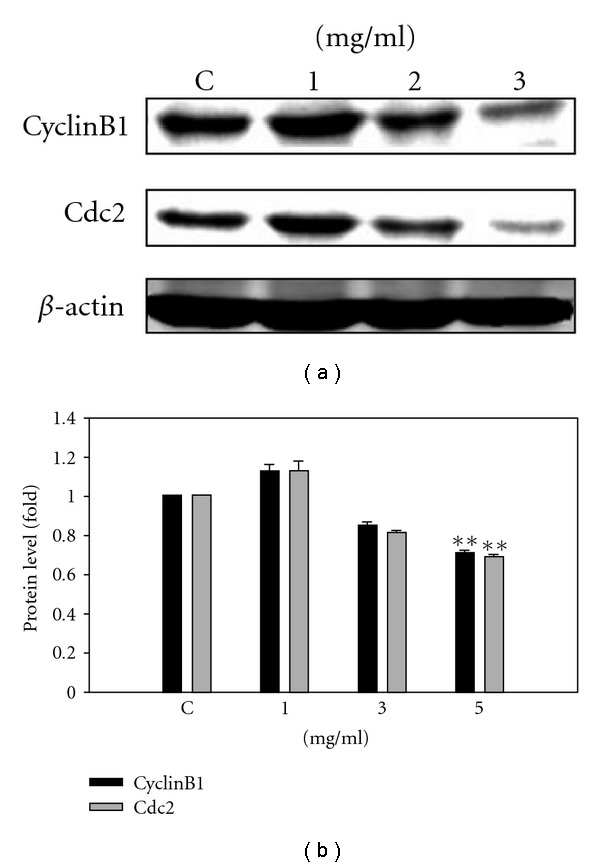
SLWLQY decreases the protein expression of G2 cell-cycle regulatory CDKs and cyclins in TSGH-8301 cells. Cells were treated with or without SLWLQY 0, 1, 3 and 5 mg ml^−1^ for 24 h. Total cell lysates were prepared and subjected to SDS–PAGE followed by Western blot, the membranes were probed with anti-cyclinB1 and Cdc2 antibody, and the results were represented by using an ECL system. *β*-actin was the loading control. Results were averaged from triplicate experiments and expressed in mean ± SD. **P < 0.01.

Using immunoblot analysis, we also observed the effect of SLWLQY treatment on the protein levels of the CDKs and cyclins during G2/M cell-cycle progression of TSGH-8301 cells. SLWLQY strongly decreased the expression of CDK1 (also known as cdc2) and cyclin B1 levels in TSGH-8301 cells (Figure 4). SLWLQY treatment at a dose of 5 mg ml^−1^ for 24 h resulted in a down regulation in CDK1 (0.7-fold) and cyclinB1 (0.72-fold) protein levels.

### 3.5. SLWLQY Induces Apoptotic Cell Death in TSGH-8301 Cells

**Figure 5:**
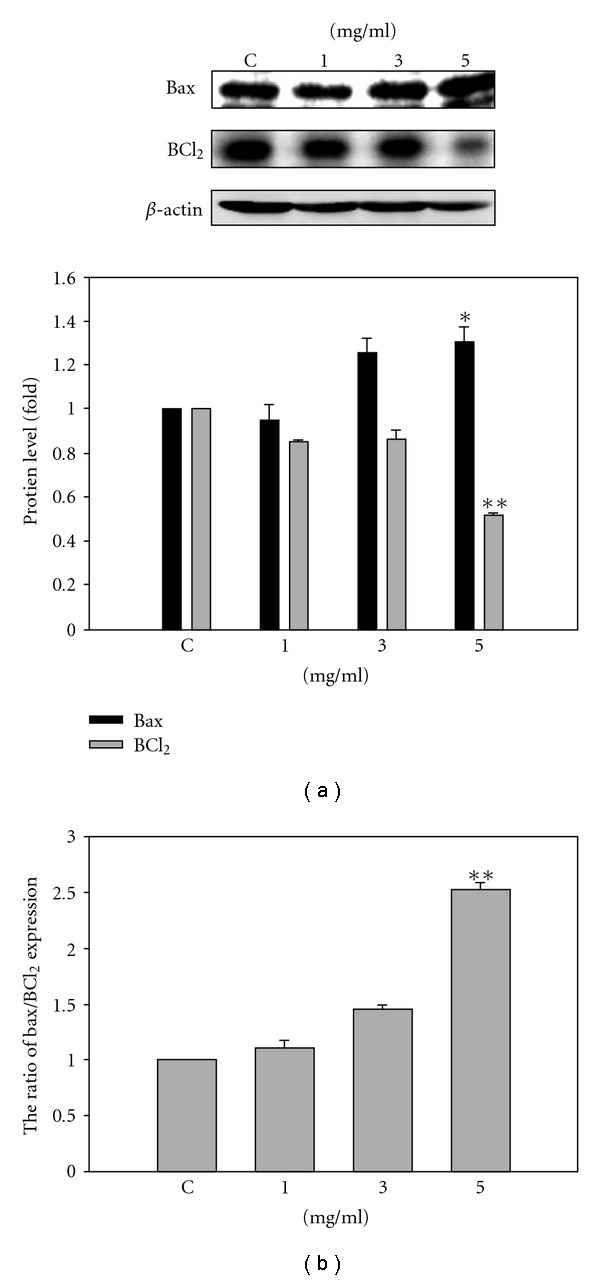
The expression of BCl_2_ family proteins (BCl_2_ and Bax) in TSGH-8301 cells treated with SLWLQY. (a) For western blot analysis, TSGH-8301 cells were treated without or varying concentrations of SLWLQY for 24 h. Membranes were probed with anti-Bcl_2_ and Bax antibody. *β*-actin was the loading control. (b) The ratio of bax/BCl_2_ expression was indicated by SLWLQY treatment. Results were averaged from triplicate experiments and expressed in mean ± SD. *P < .05, **P < .01.

**Figure 6:**
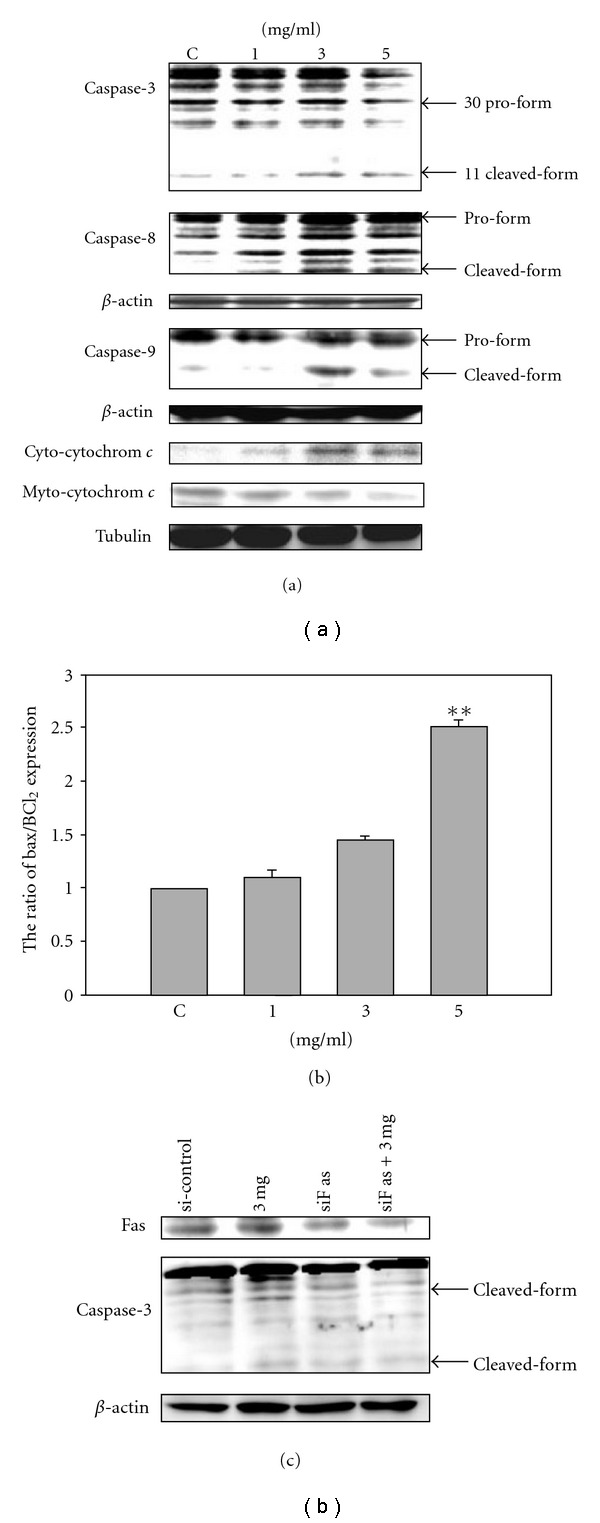
SLWLQY induced apoptotic cell death in TSGH-8301 cells. Culture cells were treated without (control) or with SLWLQY at the doses of 1, 3 and 5 mg ml^−1^ for 24 h. These proteins were detected by anti-caspases-3, caspase-8, caspase-9 and cytochrome *c* antibodies. The expression of cytochrome *c* in the cytosol and the mitochondria was assayed by immunoblotting (a) and anti-Fas/FasL antibody (b). (c) Western blot analysis of Fas protein expression and caspase-3 cleavage in cell lysates transfected with Fas siRNA and nonspecific siRNA as well as exposed to SLWLQY for 24 h. *β*-actin and tubulin were the loading control. Arrows indicated the cleavage fragment of caspases. Results were averaged from triplicate experiments and expressed in mean ± SD. *P < .05, **P < .01.

In order to assess the mechanism of SLWLQY-induced apoptosis, we evaluated the expressions of Bax, Bcl_2_, fas, fasL and caspases by western blot analysis. The expression of Bax, a proapoptotic protein, was apparently increased (1.25-fold, *P* < .05) while that of Bcl_2_, an antiapoptotic protein, was dramatically decreased by a 0.52-fold in the cells treated with 5 mg ml^−1^ of SLWLQY at 24 h (Figure 5(a)). The ratio of Bax/Bcl_2_ was increased by SLWLQY treatment (Figure 5(b)). Furthermore, caspases are cytosolic proteins that exist normally as inactive precursors with higher molecular weight (55, 32 kDa). They are cleaved proteolytically into low molecular weights (20–23 kDa) when cell undergo apoptosis [14]. In this study, the expressions of the active form of caspase-3, caspase-9 and caspase-8 were increase at the concentrations of 3 and 5 mg ml^−1^ of SLWLQY (Figure 6(a)). Next, we investigated the level of cytochrome *c* release in the cytosolic fraction following SLWLQY treatment. Cytochrome *c* is released from the mitochondrial intermembrane space, and is a key event in the activation of caspase-9 and subsequently of caspase-3 [15]. The result showed that cells exposed to SLWLQY had significantly increased cytochrome *c* in cytosolic fraction (Figure 6(a)).

In addition, activation of Fas/FasL receptor death pathway has been shown to mediate the induction of apoptosis. We next attempted to determine whether SLWLQY-mediated caspases activation was dependent on Fas/FasL activation. As shown in Figure 6(b), SLWLQY resulted in a significant increased in Fas/FasL expression from 1.00 to 1.23- and 2.1-fold, respectively (*P* < .01). Fas/FasL signaling was determined by studying the effects of Fas gene silencing. In Figure 6(c), Fas siRNA transfection largely prevented SLWLQY-induced Fas upregulation, resulting in 50% lower Fas protein levels in Fas knockdown cells compared with mock-transfected controls. Mock transfection with nonspecific siRNA did not affect Fas expression. The effect of Fas silencing on SLWLQY-induced apoptosis was assayed by western blot analysis of caspase-3 cleavage. Fas silencing resulted in a significant reduction of SLWLQY-induced caspase-3 cleavage, conforming the functional role of Fas signaling in SLWLQY-induced cell death.

### 3.6. The Anticancer Effect of SLWLQY In Vivo

**Figure 7:**
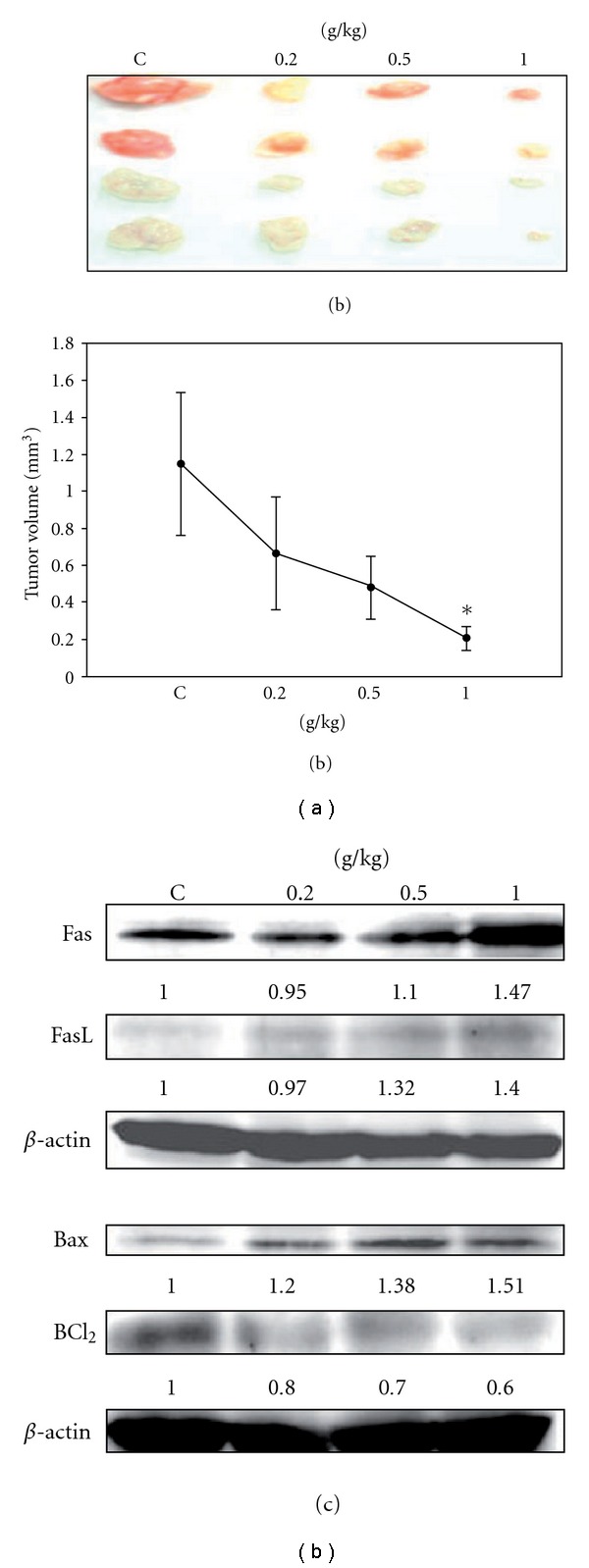
The effects of SLWLQY on the growth of TSGH-8301 cells in BALB/c nude mice. (a) TSGH-8301 cells were inoculated into nude mice and treated with SLWLQY (0.2, 0.5 and 1 g kg^−1^). The tumors were then dissected from nude mice at 4 weeks after vehicle or SLWLQY treatment. (b) Average tumor volumes of vehicle vs. SLWLQY-treated nude mice (*n* = 4) were measured at the end of experiment. (c) Western blot analyses were performed to determine the expression of Fas, FasL, Bax and Bcl_2_ proteins in the tumors. Four samples were analyzed in each group, and values represent the mean ± SD. Significance was accepted when *P* < .05. *P < .05 if compared with vehicle-treated mice.

To verify the anticancer effects of SLWLQY *invivo*, nude mice engrafted TSGH-8301 cells were treated with or without the SLWLQY (0.2, 0.5 and 1 g kg^−1^) by gavage tube. As illustrated in Figures 7(a) and 7(b), the tumor volumes of the mice treated with SLWLQY (1 g kg^−1^) were significantly reduced as compared with control, suggesting that the higher dose of SLWLQY was able to enhance tumor regression. In order to investigated the molecular mechanism that inhibited tumor growth *invivo*, the proteins lysate were extracted from tumor tissues in each group and the apoptosis-related proteins were determined by western blotting analysis. We found that the expressions of Fas, FasL and Bax proteins in tumor tissues were elevated in the groups treated with SLWLQY. In contrast, the expression of Bcl_2_ was down-regulated in the SLWLQY-treated tumor tissues (7(c)).

## 4. Discussion

While many anticancer agents have been developed and used, the side effects and resistance are serious problems have to be overcome in the treatment of cancer [16]. Therefore, development of better therapeutic drugs has become necessary. SLWLQY is characterized by the use of mixtures of 16 herbs in a single formula. The molecular mechanism on anti-cancer effects of SLWLQY is not yet elucidated. It has been shown that SLWLQY contains many polyphenolic compounds, such as gallic acid (2.28%), PCA (0.4%), EGCG (0.46%), caffeic acid (1.46%), rutin (6.71%), quercetin (1.43%) and unknown polyphenols (Figure 1 and Table 2). Phenolic or flavonoid compounds possess strong anticancer and antioxidant properties [17, 18]. Agarwal et al. demonstrated that gallic acid could induce G2/M phase cell-cycle arrest and apoptosis in human prostate carcinoma DU145 cells. These findings suggested that in SLWLQY, polyphenols could be bioactive compounds. In fact, our unpublished data indicated that gallic acid could cause both G2/M phase arrest and apoptosis in TSGH-8301 cells.

In this study, SLWLQY inhibited the growth of Huh-7, AGS, KB, MCF-7, WRL68 and TSGH-8301 cells through the induction of apoptosis (Figure 2). Among the cancerous cell lines, SLWLQY possessed the strongest potency to induce cytotoxicity in TSGH-8301 (human bladder carcinoma cells). However, WRL68 was less sensitive to cytotoxic effect of SLWLQY than the other cancer cells, indicating that tumors cells are more responsive to the SLWLQY treatment. We also have shown that SLWLQY-treated TSGH-8301 cells are arrested irreversibly in the G2/M phase of the cell cycle (Figure 3). In addition, no change of S phase was observed. Cell-cycle arrest in SLWLQY-treated cells was accompanied by a marked declined in the level of cyclinB1 and Cdk1 (Cdc2) (Figure 4). It is reasonable to postulate that SLWLQY treatment may affect activity of Cdk1/cyclin B kinase by reducing cdk1-cyclin B1 complex formation. More specially, Cdc2 could be phosphorylated at Thr 14 and Tyr 15 by protein kinase, such as Myt1 and Wee1, then converted into an inactive precursor. Whereas, Cdc2 could be also dephosphorylated by Cdc25c and becomes inactive [19, 20]. However, more studies are needed in the future to further support this assumption, as well as to define the role of upstream events such as CDKI, Chk1 and Chk2 and their regulators ATM and ATR in the observed G2/M phase arrest by SLWLQY in TSGH-8301 cells.

In recent years, evidence has suggested that apoptosis is important to most anticancer agents in tumor cells [21, 22]. There are two distinct pathways that initiate apoptosis designated as mitochondrial and receptor death pathways [23, 24]. The mitochondrial death pathway is controlled by members of Bcl_2_ and Bcl-XL proteins and the proapoptotic Bax and Bid proteins [25, 26]. The proto-oncogene Bcl_2_ encodes an inner mitochondrial protein that reportedly antagonizes apoptosis in many tumor cells [27]. SLWLQY-induced apoptosis was found to be associated with the downregulation of Bcl_2_ (Figure 5). On the contrary, overexpression of Bax accelerates apoptotic death induced by different stress in many cell lines. Our founding showed that TSGH-8301 cells expressed high level of Bax after SLWLQY treatment to facilitate the induction of apoptosis.

For evaluation of the death receptor pathway, the roles of Fas and FasL are well-characterized members. Engagement of Fas by a FasL led to the formation of a protein complex and permit execution of apoptosis by caspase-8 activation [28]. In this study, an increase of Fas and FasL protein was observed in the SLWLQY-treated cells (Figure 6(b)). Release of FasL initiated apoptosis through Fas death receptor which subsequently activated caspase-8 (Figure 6(a)) and cytochrome *c* implying an association between the death receptor pathway and SLWLQY-induced apoptosis. Moreover, the apoptotic death receptor pathway and mitochondrial death pathway could converge on activation of caspase-3. Finally, the direct link between Fas/FasL up-regulation and SLWLQY- induced apoptosis was provided by Fas gene silencing using RNA interference strategies [29]. Fas silencing resulted in a significant reduction of caspase-3 cleavage by SLWLQY in TSGH-8301 cells. In our study, SLWLQY induced the activation of Bax from the mitochondria and caspase cascade activation (Figures 5 and 6). These implied that SLWLQY triggered the signaling to induce cell apoptosis via both mitochondrion- and death receptor-mediated pathways. In addition, our *invivo* study demonstrated that the apoptosis-related proteins (Fas/FasL, Bax/Bcl_2_) were also changed in the xenografed tumors. We also showed evidence that the antitumor activity of SLWLQY treatment against TSGH-8301 cells xenografted in nude mice (Figure 7). Treatment with the highest doses of SLWLQY (1 g kg^−1^) did not decrease the body weight when compared to the control mice.

In summary, our findings demonstrating significant G2/M phase arrest and apoptosis by SLWLQY, suggest that SLWLQY can exert anticancer activity in human bladder cancers. However, more studies are necessary to clarify the mechanism of SLWLQY or its isolated bioactive compounds in the near future.

## Funding

National Science Council (94-2320-B-040-038) in Taiwan; the Chung Shan Medical University Research Fund (CSMU 92-OM-B-011 and CSMU 92-OM-B-034).

## Figures and Tables

**Table 1:** The composition of SLWLQY.

Scientific name	Grams
(1) *Panax Ginseng* C.A. Mayer	50
(2) *Astragalus Membranaceus*	50
(3) *Glycyrrhiza Uralensis Fisch*	12.5
(4) *Angelica Sinensis* (Oliv.) Diels	50
(5) *Paeonia lactiflora Pall*	50
(6) *Aucklandia lappa Decne*	20
(7) *Citrus aurantium L*.	25
(8) *Areca catechu* Linn	12.5
(9) *Cortex Magnoline Officinalis*	20
(10) *Linderae Radix*	20
(11) *Cinnamomum cassia presl*	20
(12) *Saposhnikovia divaricata* (Turcz.) Schischk	25
(13) *Perilla frutescens* (Linn.) Britt.	25
(14) *Angelicae Dahuricae Radix*	25
(15) *Ligustici Rhizoma*	25
(16) *Platycodon grandiflorum* A. DC	25

**Table 2:** Characterization of phenolic compounds of SLWLQY.

Assigned identity	SLWLQY (mg g^−1^)
Gallic acid	228
PCA	40
EGCG	46
Caffeic acid	146
Rutin	671
Quercetin	143
